# A comparative guide to expression systems for phage lysin production

**DOI:** 10.1042/EBC20240019

**Published:** 2024-12-17

**Authors:** Emma Cremelie, Roberto Vázquez, Yves Briers

**Affiliations:** 1 Laboratory of Applied Biotechnology, Department of Biotechnology, Ghent University, Ghent, Belgium; 2 Centro de Investigación Biomédica en Red de Enfermedades Respiratorias (CIBERES), Madrid, Spain

**Keywords:** Endolysin, Expression, Host selection, Phage lysin, Recombinant protein

## Abstract

Phage lysins, bacteriophage-encoded enzymes tasked with degrading their host’s cell wall, are increasingly investigated and engineered as novel antibacterials across diverse applications. Their rapid action, tuneable specificity, and low likelihood of resistance development make them particularly interesting. Despite numerous application-focused lysin studies, the art of their recombinant production remains relatively undiscussed. Here, we provide an overview of the available expression systems for phage lysin production and discuss key considerations guiding the choice of a suitable recombinant host. We systematically surveyed recent literature to evaluate the hosts used in the lysin field and cover various recombinant systems, including the well-known bacterial host *Escherichia coli* or yeast *Saccharomyces cerevisiae*, as well as plant, mammalian, and cell-free systems. Careful analysis of the limited studies expressing lysins in various hosts suggests a host-dependent effect on activity. Nonetheless, the multitude of available expression systems should be further leveraged to accommodate the growing interest in phage lysins and their expanding range of applications.

## Introduction

During their lytic cycle, bacteriophages must cross the bacterial cell wall twice. For these endeavours, they are equipped with phage lysins: enzymes specialised in the degradation of peptidoglycan (PG), creating either local perforations or causing full cell lysis [1]. Interestingly, when applied exogenously in purified form, recombinant lysins can effectively and specifically lyse pathogenic bacteria, making them potent antibacterial candidates [2–4]. They provide a vital response to the concerning threat of antimicrobial resistance menacing global healthcare, where many traditional antibiotics are rendered ineffective due to massive over- and misuse [5]. Indeed, lysins have been proven successful against multidrug-resistant pathogens in several *in vitro* and *in vivo* models and are considered one of the most clinically advanced new classes of antimicrobials [6,7]. Besides their use in medicine, phage lysins are now investigated for a wide range of applications, including agriculture [8], food safety [9], and biotechnology [10]. This increasing interest stems from their unique features including rapid action, non-toxicity, tuneable specificity, low probability of resistance development, and wide abundance in nature [11].

**Figure 1:**
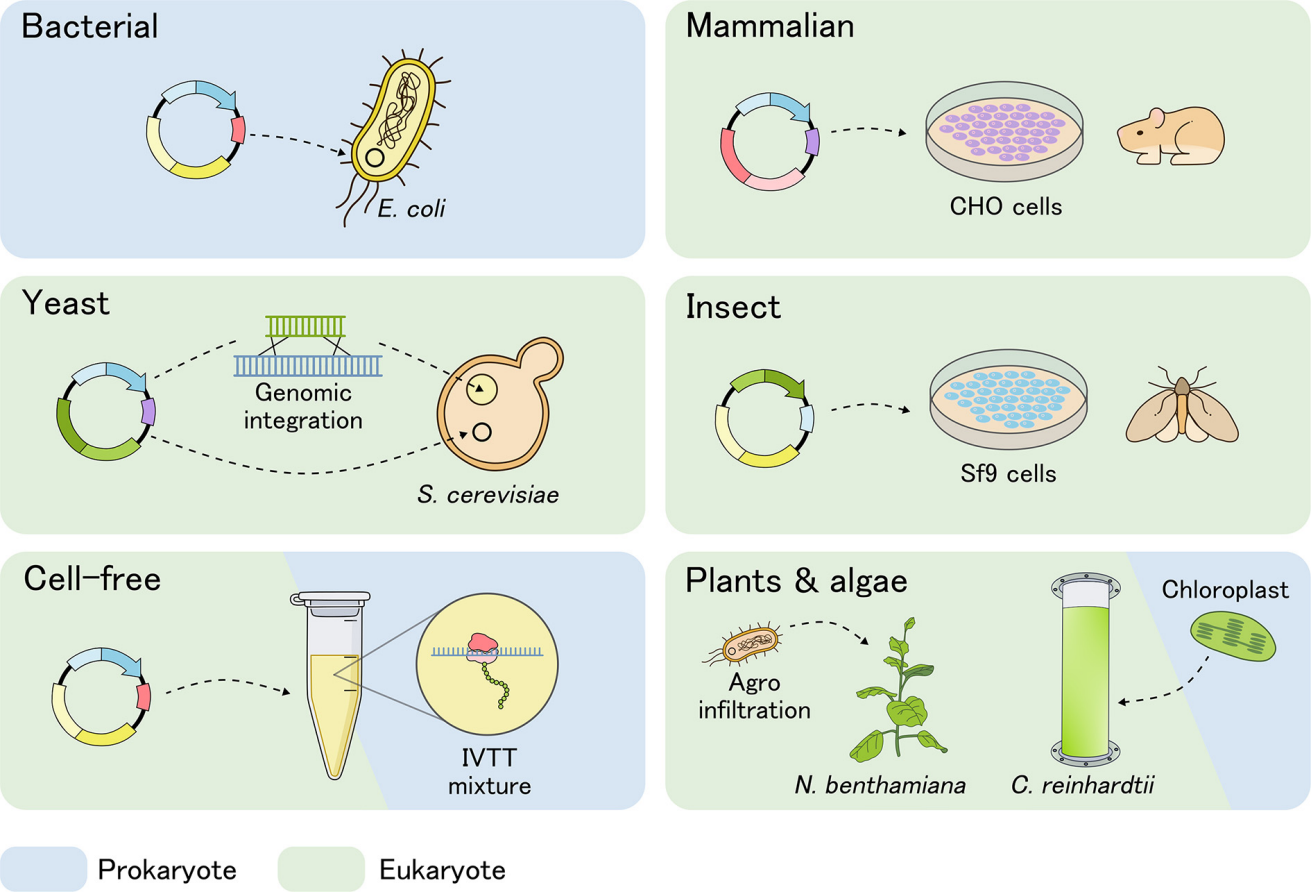
Different recombinant expression platforms and their methods Each group of host cell types depicts the most frequently used methods. Groups are coloured by their respective cell organization. For bacterial or yeast expression (e.g. in *E. coli* or *Bacillus* spp., and *Saccharomyces cerevisiae* or *Pichia pastoris*), plasmid-based expression is available, whereas stable genomic integration is frequently used in the latter as well [19]. Both mammalian and insect expression can be performed using transfected cell lines in suspension cultures. For example, Chinese hamster ovary (CHO) or HEK293 (human) mammalian cell lines, or Sf9 or Sf21 insect-derived ones. Mammalian expression can be mediated transiently or with stable cell lines for small and large-scale expression, respectively [20]. For insect expression, baculovirus vector systems are the most common [19]. Cell-free expression can be achieved using *in vitro* transcription-translation (IVTT) mixtures derived from cell extracts of, e.g., *E. coli* or CHO cells, aimed for expression in either prokaryotic or eukaryotic environments [21]. For expression in plants such as *Nicotiana benthamiana* or *Nicotiana tabacum*, both transient or stable expression is possible. Introduction of genetic material can be mediated by agroinfiltration, in which the desired gene is transferred into the plant cells by *Agrobacterium tumefaciens* [22]. Expression in a prokaryotic-like environment is also possible in the chloroplasts of green algae (e.g. *Chlamydomonas reinhardtii*), as this cell organelle evolved from a Gram-negative cyanobacterial ancestor through the process of endosymbiosis, later losing its PG layer [23]. Such algal expression hosts can be cultured in photobioreactors [24].

**Figure 2:**
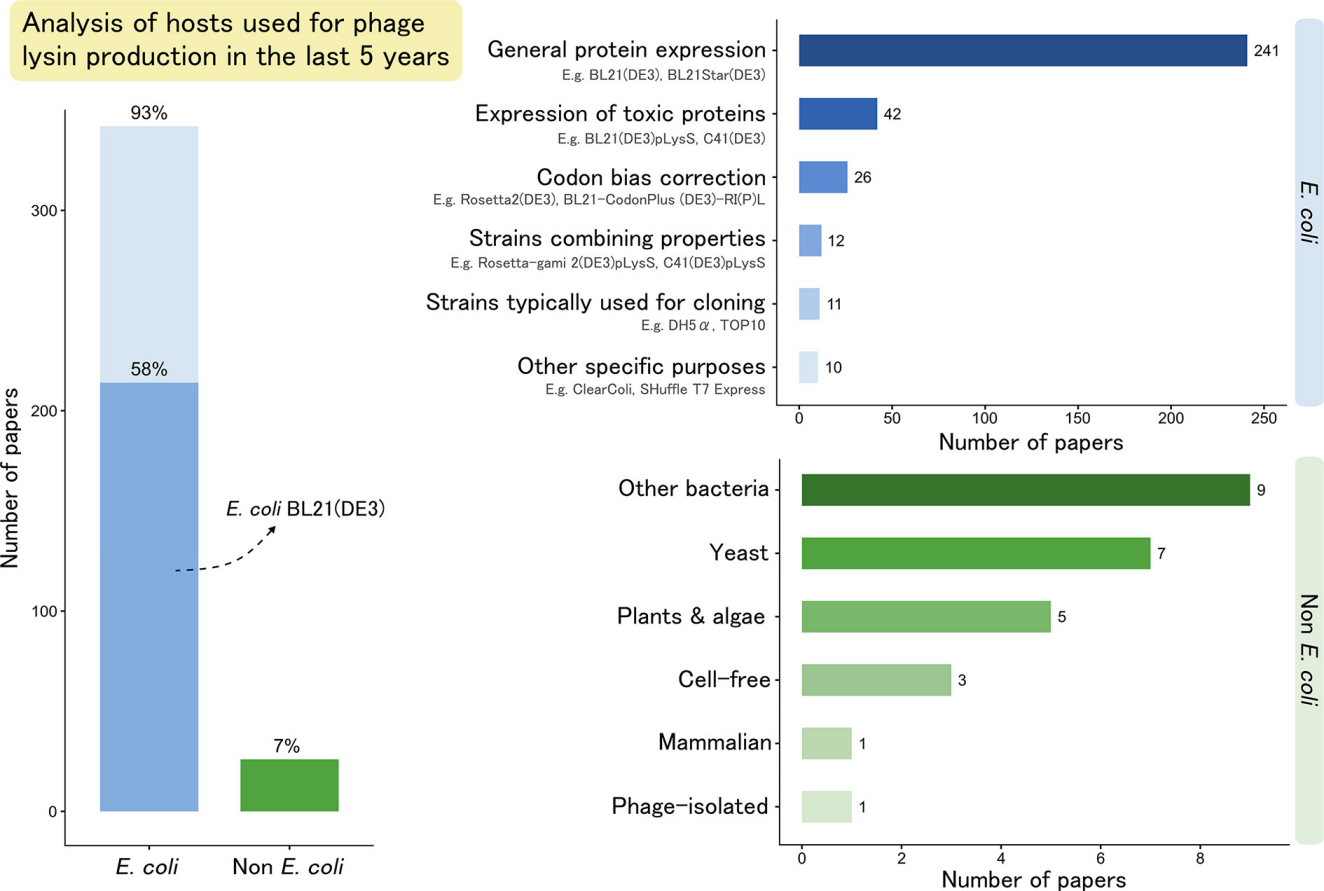
Distribution of expression hosts used in phage lysin-related literature between 2019 and 2024 Literature searches were performed on April 12, 2024, from relevant databases (Web of Science, PubMed, ScienceDirect, and Scopus) in, when available, the article title, abstract, keywords, or MeSH terms. The used search query was (‘Lysin’ OR ‘Endolysin’) AND (‘Phage’ OR ‘Bacteriophage’), as these were previously found the most popular names given to the discussed enzymes [25]. Obtained articles (643) were filtered for relevance based on their title and abstract, and the remaining papers (453) were read to extract the expression host used.

Most Gram-positive phage lysins have a modular structure, consisting of one or more enzymatically active domains and a cell wall-binding domain [12]. While the former provides cleavage of specific bonds within the PG, the latter ensures recognition and binding of the protein to its substrate. This modularity, alongside the wide variety in lysin domains, has been extensively leveraged by protein engineers to modulate lysin activity, specificity, and stability [12–15]. Also for Gram-negative pathogens, the PG can be targeted by specific addition of modules to penetrate their outer membrane [16]. These engineering efforts are enabled by recombinant DNA technology, allowing the heterologous production of lysins in naturally non-producing but easy-to-manipulate cells [17]. Numerous recombinant expression platforms have been developed, each with its own features (Figure 1). According to our survey of lysin-related literature in the last 5 years, 93% of research is performed using typical *Escherichia coli* strains, with 58% accounted for by BL21(DE3) (Figure 2). This aligns with the host-decision tree proposed by Schütz et al., which recommends the production of phage lysins in bacterial systems such as *E. coli* [18]. However, various other systems have been explored for phage lysin production across different applications. Nevertheless, it is important to recognize that different hosts may yield proteins with different properties, and hence, several considerations should be made when expressing phage lysins.

This mini-review focuses on the assorted hosts available for lysin expression, providing an overview of the necessary key considerations, driven by different applications. Additionally, we review comparative studies to assess the potential impact of the expression host on lysin activity and yields. We refer the interested reader to other reviews [19–22, 26–28] for thorough background information on the described expression hosts. For clarity, we will use the term ‘(phage) lysin’ to encompass both natural virion-associated lysins and endolysins, or their engineered variants [25]. With this work, we aim to guide aspiring lysin researchers in their informed selection of the appropriate host, while giving experienced practitioners a peek into the underexplored potential of alternative expression platforms.

## Key considerations when selecting a host for phage lysin expression

The choice of a suitable host for lysin production may involve considering the differential features of the available systems, as summarized in Table 1. General and practical considerations such as lab expertise and availabilities, production cost, time, and effort, ease of purification, ease of optimization, and scalability are relevant when making a choice on the appropriate lysin expression host. Also, the specific application drives this host choice, as discussed in the next section. Besides these, several specific host-dependent considerations, to which special attention has been paid in literature, can be crucial in the lysin context and are discussed below.

**Table 1:** Overview of recombinant expression hosts for phage lysin production, their respective features in this context and reported lysin yields

Cell type	Expression environment	Host features [19,29]		Hosts used for lysin expression	Reported lysin yields	References
		Advantages	Disadvantages			
Bacterial	Prokaryotic	- Well-established - High yields - Low cost - Rapid - No PTMs	- Endotoxin contamination (Gram-negatives) - Inclusion body formation	*Escherichia coli* BL21(DE3)	1.6 mg/L of LYZ2 – 3323 mg/L of TSPphg	[30,31]
				*Lactococcus lactis* NZ9000 or MGI363	N/A	[32,33]
				*Lactobacillus johnsonii* FI9785	N/A	[34,35]
				*Lactobacillus casei* BL23	N/A	[36]
				*Lactobacillus plantarum* ATCC 14917	∼ 10 µg/L of Ply511	[37]
Yeast	Eukaryotic	- High yield - Low cost - High cell density growth - Often GRAS - No endotoxins - Protein folding quality control	- PTMs	*Saccharomyces cerevisiae* D452-2 or EBY100	N/A	[38,39]
				*Pichia pastoris* GS115	239 µg/mL final concentration of purified LySP2	[40]
				*Hansenula polymorpha* NCYC495	43 mg/L of PVP-SE1gp146	[41]
Plants and algae	Eukaryotic or prokaryotic-like within the chloroplasts	- No endotoxins - Often GRAS - Low cost - Protein folding quality control - High scalability	- Low yields - Slow - Stable line production difficult and time-consuming - PTMs	*Chlamydomonas reinhardtii* TN72 (chloroplast-based expression)	∼1 mg/L culture volume or ∼1.3 mg/g algal dry weight of both Pal and Cpl-1	[42]
				*Nicotiana tabacum* cv. Petite Havana (chloroplast-based expression)	∼0.5 mg/g fresh weight of Cpl-1 and ∼2 mg/g fresh weight of Pal	[43]
				*Nicotiana benthamiana*	550 µg/g fresh weight of ZP173 or 100 µg/g fresh weight of PlyCP41	[44,45]
Mammalian	Eukaryotic	- No endotoxins - Protein folding quality control	- High cost - Low yields - PTMs - Low scalability	Human cell lines HEK293T, A549, and HepG2	N/A	[46]
Insect	Eukaryotic	- No endotoxins - Protein folding quality control	- High cost - Low yields - PTMs	N/A	N/A	N/A
Cell-free	Eukaryotic or prokaryotic	- No endotoxins - Versatility - No host toxicity	- High cost - Low yields - Low scalability	Extract of *Escherichia coli* slyD	N/A	[47]

Some features may function as both advantages or disadvantages, depending on the envisioned application. Examples of reported lysin yields using a specific host organism are given and are expressed in terms of mg of recombinant (purified) protein per litre of expression host culture, unless stated otherwise. N/A indicates instances where, to the best of our knowledge, expression of lysins in this cell type has not yet been attempted, or yields were not reported.

### Endotoxins

Accounting for endotoxin (or lipopolysaccharide, LPS) contamination of the recombinant lysin preparation can be a good reason to select one host over another. Such toxins originate from the outer membrane of Gram-negative hosts, which is why these can be particularly relevant when using *E. coli* for lysin expression [48]. As endotoxins can elicit inflammatory responses or even septic shock when administered systemically, they must be avoided or removed in the case of therapeutic proteins [49,50]. A variety of routine strategies are available for this task, such as anion-exchange chromatography or washing with detergents [51]. Alternatively, the engineered *E. coli* strain ClearColi is commercially available for endotoxin-free *E. coli* expression, as it is deficient in the specific endotoxic LPS molecules yet allows for high-yield protein production [52]. Though this strain is ideal for lab-scale experiments, several other potentially immunogenic bacterial components might remain in the derived protein preparations [49]. Therefore, alternatives for avoiding endotoxins may include changing the expression host to inherently endotoxin-free ones, for example using Gram-positive hosts (*Bacillus* spp. or lactic acid bacteria, LAB), or eukaryotic hosts such as the Generally Regarded As Safe (GRAS) *Saccharomyces cerevisiae* [53].

### Glycosylation

Glycosylation is a post-translational modification (PTM) in which sugar moieties are covalently bound to the protein backbone. It is most common in eukaryotic cells, where it is associated with the protein-folding machinery [54–56]. The attached sugar profiles differ between expression hosts. For example, N-linked glycoproteins produced in *S. cerevisiae* are often hyper-mannosylated [57], while plant glycans are modified with fructose and xylose sugars [58], both differing from the native N-glycan structures found in human glycoproteins. When glycosylation of heterologous proteins occurs, it can lead to problems such as an immune response and clearance of therapeutic proteins from the bloodstream due to the detection of non-human glycosylation patterns [59], or in general, an alteration of the protein properties related to their therapeutic action [60]. Among lysins, it has been shown that N-linked glycosylation of both endolysin Cpl-1 and bacteriocin lysostaphin results in a reduced activity [46,61]. For Cpl-1, a killing activity of 2.58 log units was observed for the non-glycosylated enzyme, compared with 1.92 using its glycosylated variant. O-glycosylation has even been described as an activity-regulation mechanism for an autolysin of *Lactiplantibacillus plantarum*, since removal of its O-glycans was proven to increase its activity [62]. As phage lysins are naturally produced in prokaryotic environments [1], their activity does not rely on eukaryotic PTMs and are hence unnecessary or even detrimental. Indeed, it can be reasoned that modification of the critical amino acid regions responsible for catalytic activity or substrate binding might hamper their functions. Besides, other mechanisms may lead to glycosylation-mediated inactivation of lysins, such as a change in the lysin surface properties by providing additional negative charges, or steric hindrance by the added glycans. These effects are however in contrast with most human therapeutic proteins, which often require glycosylation for correct activity and are therefore typically produced in mammalian cells [63]. Nonetheless, it is clear that avoiding glycosylation is usually desired when expressing lysins, which could be achieved by using prokaryotic hosts, but also by expression in specific organelles of eukaryotic hosts, such as the chloroplasts of plants and algae or the mitochondria of yeast [42,64].

On the other hand, glycosylation of less essential protein regions might enhance other lysin properties. Introduction of N-linked glycan sites has been shown to increase the serum half-life of human growth hormone as a therapeutic protein, while removal of glycans has shown the opposite for erythropoietin and interferon α [65–67]. Additionally, the solubility of therapeutic proteins has been increased with glycoengineering, and human-type glycans could also be employed in masking strategies to avoid immune responses [68,69]. As such, deliberate glycosylation of phage lysins might be useful to consider for modulation of their pharmacokinetics. To these ends, several engineering efforts have altered expression hosts such as *E. coli* and *Pichia pastoris* to allow production of human-type glycoproteins, although these are not yet widely tested for lysin expression [28,63,70,71].

### Solubility and stability

Recombinant (over)expression in bacterial systems such as *E. coli* is often plagued by the aggregation of insoluble protein in dense particles, termed inclusion bodies. While many processes can lead to protein aggregation, inclusion body formation is often related to the misfolding of heterologous proteins [72]. A possible reason for the formation of inclusion bodies can be a difference in codon usage between the protein’s original and recombinant host, leading to a differential impact on translation rate [72,73]. Several strategies exist to isolate and refold proteins from inclusion bodies, yet final yields are often low or non-existent [74]. Thus, host engineering has been aimed to reduce inclusion body formation. For example, co-expression of rare tRNAs in *E. coli* strain BL21-CodonPlus (DE3)-PI(P)L [27,75], or methods like codon optimization and the more advanced algorithm of codon harmonization, which can be applied to ameliorate codon bias at the gene design stage [76–78].

Also the lack of proper disulfide bond formation when expressing proteins in the reducing cytoplasm of *E. coli* could, in principle, result in misfolded proteins [72]. While, to the best of our knowledge, no natural lysins with essential disulfide bonds exist, their introduction could be beneficial for lysin expression or performance. For example, dimerization of lysin Cpl-1 by the introduction of disulfide bonds has been shown to increase both its activity and plasma half-life [79]. In contrast, disulfide bonds can act as negative regulatory elements for lysin activity, such as the ‘disulfide caging’ phenomenon in some SAR-endolysins [80,81]. Disulfide bonds can also be detrimental in engineered lysins, such as Art-085, where mutation of its cysteine residues and subsequent removal of disulfide bonds increased its thermostability [82]. Hence, introduction of disulfide bonds in lysins may be either beneficial or detrimental depending on the application, and thus, the expression host should be chosen accordingly. To facilitate disulfide bond formation in the *E. coli* cytoplasm, strains like SHuffle or Origami have been developed [83]. Also trafficking to the periplasm of *E. coli* using signal peptides can aid disulfide bond formation, as this cell compartment harbours endogenous disulfide isomerases [63]. However, the latter option might be less favourable for phage lysin production due to toxic effects, as the lysins’ PG substrate is present within this cell compartment. Lastly, expression in eukaryotic hosts such as yeast, plants and their chloroplasts, or mammalian cells allows for the formation of disulfide bonds [19,84].

### Toxicity

Finally, toxicity of genes or gene products is a common phenomenon leading to failure of recombinant protein production [85]. Such toxic effects when using *E. coli* for lysin expression may arise through an interaction of the produced lysins with cell wall elements of its expression host. For example, as mentioned, if the expressed lysins reach the hosts’ periplasm, catalytic degradation of its PG could occur. Besides, other interactive phenomena hampering efficient lysin expression could be imagined, such as a too high affinity of the cell wall-binding domain towards the host PG, in a way that the expressed proteins remain bound to the host after cell disruption. Such effects would reduce the yield of lysins that could eventually be recovered; however, these phenomena are not well investigated. Nevertheless, some generic solutions exist to prevent toxicity due to gene expression. For example, *E. coli* BL21(DE3)pLysS could be used, which reduces basal expression by co-expressing the T7 lysozyme [86]. Alternatively, hosts without PG such as plants or yeasts might alleviate the described problems [23]. Indeed, several studies report failed expression of Gram-negative lysins in *E. coli*, subsequently and successfully resorting to such other expression hosts [40, 87–89]. Lastly, an expensive but appropriate solution for toxic protein production are cell-free expression systems [21].

## Application-driven examples of phage lysin expressions

**Figure 3:**
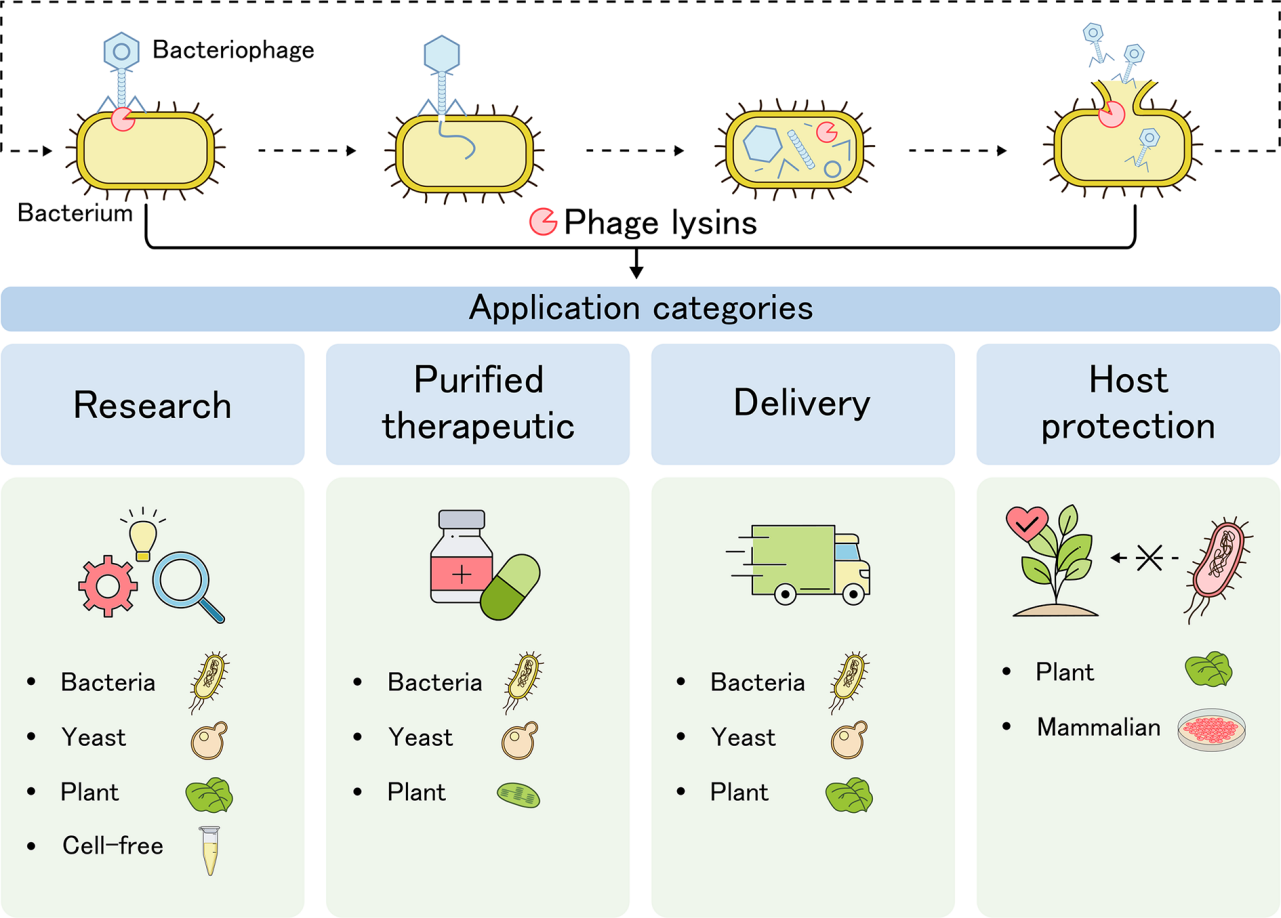
Overview of recombinant expression hosts for various categories of phage lysin applications The upper panel depicts the role of lysins in the phage lytic cycle, starting with phage attachment to the host and local PG degradation by virion-associated lysins, allowing injection of genetic material. Following synthesis of new phage genetic material and proteins, virions are assembled and released by host cell lysis, in part mediated by endolysin muralytic activity. Released viral progeny can now infect new host cells, restarting the lytic cycle [1]. The lower panel depicts the different categories of applications in which lysins are used and gives examples of which host cells have been investigated for these purposes.

Current proposed applications for the heterologous expression of phage lysin are diverse, but fall into four categories (Figure 3): expression for research purposes, for the production of purified therapeutics, for lysin delivery, and for protection of the host itself. Considering that each category requires specific properties, different hosts with suitable features, as dictated by the application, have been tested and are discussed below.

### Expression hosts for lysin research

A first category of applications is research purposes, specifically for the discovery and engineering of phage lysins using screening assays [90]. Here, high yield but mainly throughput and time consumption are key factors to take into account. Additionally, specific screening assays may call for specific host characteristics. Within this category, either purified or non-purified proteins are used to evaluate the activity, specificity, stability, etc., using a variety of assays.

In the case of purified proteins, typical assays are turbidity reduction (TRA), minimum inhibitory concentration (MIC), or growth inhibition assay [91]. An important concept to introduce is ‘you get what you screen for’, meaning that there is a strict dependence of the screening output on the conditions of the screening itself (assay set-up, pH, buffer composition, temperature, etc.) [92]. Hence, screening should be conducted under relevant conditions, set according to the research question or applied aim. When screening for activity, one is often screening for high expression as well, which is desirable in most contexts. However, if screening is performed in host A with the intention of eventually producing it in host B, the (high) expression levels may not necessarily translate between hosts. A general recommendation is thus to screen using the expression host envisioned for the final application. For research purposes, the *E. coli* expression platform is generally the first choice for lysin production, as demonstrated by our survey (Figure 2). It is well-established and the necessary molecular tools ranging from gene cloning to protein purification are available in most biotechnology research labs [27,75]. Nevertheless, several alternative expression systems have been used for screening. For example, research-grade lysins can be produced in *P. pastoris*, *S. cerevisiae*, or even plants [87,93]. The former two deliver recombinant lysins quickly and allow translation to high throughput. Furthermore, cell-free systems can produce recombinant proteins in small scale using *in vitro* transcription-translation (IVTT) mixtures containing cell extracts of virtually any host, including *E. coli*, yeast, Chinese hamster ovary (CHO) cells, insects, or plants [21]. Here, the gene of interest is expressed by the cellular components of the added extracts, allowing rapid and specific production of the protein when cultivation is not possible or desirable [21]. Such a set-up was recently investigated using *E. coli* extracts for screening of chimeric lysins targeting *Staphylococcus aureus* [47].

On the other hand, when purified proteins are not needed, several more creative assays are available. For example, the plate lysis assay is a common activity assay with a high throughput. While various protocols exist, one version involves combining the expression inducer with autoclaved bacterial cells in a plate, on which the lysin-producing host is spotted [94]. In this configuration, *E. coli* BL21(DE3)pLysS is particularly interesting to use, as it is hypothesized that the co-expression of T7 lysozyme leads to cell death or increased permeabilization, enhancing the release of lysins and facilitating the formation of a halo zone [94]. However, this might in turn lead to false positive results of the T7 lysozyme itself. Another assay recently evaluated for lysin screening is the self-depletion assay [95]. Here, the lysin variants are expressed in such a way that active variants kill the expression host, while non-active variants have minimal impact on host viability. Hence, active lysins are depleted, and non-active ones are enriched, which can both be identified by sequencing the initial and final cultures. While only *E. coli* has been used as expression host in this set-up, applications could be envisioned where the originating phage- or lysin-targeting bacterium is used. Lastly, another inventive assay is the high-throughput protease stability assay based on *S. cerevisiae* yeast surface display [96]. In the latter technique, lysins are intracellularly expressed and anchored outside to the yeast cell wall, where they can be evaluated with flow cytometry for properties such as stability [97]. This assay has recently been successful in increasing the stability of lysins LysEFm5 and LysCP2 [95,98].

### Expression hosts for therapeutic lysin production

A second category is hosts for obtaining purified phage lysins as therapeutic proteins. As with research purposes, high yields of soluble protein but also low cost, safety, and scalability are central features here. Expression in *E. coli* strains is again the most common platform considered for this purpose, as it is extensively researched, highly developed, and optimized. Several submitted patents (e.g. by Micreos and ContraFect) concerning the development of lysins as therapeutics also state the use of an *E. coli* expression system as the first preference, while recent research focuses on further process scale-up using this host [31,99,100]. However, the safety aspect due to endotoxins and the associated necessary purifications remain a challenge of this bacterial expression system [101]. Alternatively, production in yeast has shown high recombinant protein yields (Table 1). While not attempted for purified therapeutic phage lysins, *P. pastoris* has been investigated to produce antimicrobial lysozymes from several sources [102–104]. Also, yeast *Hansenula polymorpha* has recently been explored for this purpose with comparable yields of endolysin PVP-SE1gp146 as produced by *E. coli* [41]. Furthermore, several transient plant expression systems using *Nicotiana* spp. have been explored for large-scale production of lysins specific for *Streptococcus pneumoniae*, *Clostridium perfringens*, and *S. aureus* [44,45,93,105]. However, while these plant platforms are low-cost and carry the GRAS status, their transient character does not suit the requirements for commercial production well. Instead, stable expression using the chloroplasts of either *Nicotiana* spp. or *Chlamydomonas reinhardtii* algae could serve this purpose better. They have both been used for the successful expression of anti-pneumococcal endolysins Cpl-1 and Pal [42,43]. The algae system is particularly attractive since the synthesis of recombinant proteins is light-driven, and allows for cost-effective and scalable production in photobioreactors [24]. A patent for the large-scale expression of an endolysin against *Propionibacterium acnes* using cyanobacteria has also been published [106]. Nevertheless, such production processes may require investments in specific equipment and expertise, unlike the use of universal production fermenters.

### Expression hosts for lysin delivery

A third category is where the expression host is intended as a delivery vehicle. Here, specific host characteristics, such as probiotic properties or food safety are of interest. Examples include uses in therapy, food production, agriculture, and industry.

Several phage lysins have been investigated for targeting bacterial pathogens in the gastrointestinal tract (GIT) due to their high specificity and hence, likely microbiome friendliness [107]. However, successful delivery via oral administration remains a challenge due to in-transit degradation by digestive enzymes or the low pH [108]. Designer probiotics engineered to express phage lysins have been explored as a potential solution [69,109]. Several LAB strains have proven probiotic properties and are GRAS-classified, making them ideal candidates for GIT delivery [110]. Though, it should be noted that the GRAS status is not retained after genetic engineering of these strains needed for expression of lysins, and a new certification of the GRAS label should be obtained. Nevertheless, Gram-positive LAB including *Lactococcus lactis* strains and *Lactobacillus johnsonii* FI9785 have already been evaluated for the expression and secretion of lysins targeting Gram-positive *S. aureus*, *C. perfringens*, and *Listeria monocytogenes* [34,35,37,111], as well as various Gram-negative pathogens [89]. While extensively tested *in vitro*, *in vivo* evaluation of the effectiveness and microbiome-editing properties of these lysin-producing strains is, however, still lacking. Similarly, active and stable expression of an endolysin targeting *S. aureus* in yeast *S. cerevisiae* EBY100 using surface display has been successful [39], which could be extended to proven probiotic yeast strains for therapeutic purposes [112].

For food applications, *L. lactis* strains and *Lactobacillus casei* BL23 have been engineered for the production of lysins against common spoilage bacteria *Clostridium tyrobutyricum* [113], *L. monocytogenes* [33], and *S. aureus* [36,114] to be used in the dairy industry. Lysin expression in edible plants has also been proposed as an additive in human food or animal feed to eliminate or prevent food pathogens [44,45,105]. In addition, *P. pastoris* strain X-33 has been evaluated *in vitro* as a probiotic oral delivery method for lysins in both aquaculture and poultry [40,115]. Also expression host *C. reinhardtii* is currently being explored by the company Axitan for lysin delivery in poultry [116]. Lastly, yeast *S. cerevisiae* and *P. pastoris* have been tested in various set-ups for the production of lysins targeting ethanol fermentation contaminants [38,117,118].

### Lysin expression for host protection

A final but currently limited category is those applications where the host itself needs to be protected, achieved by its own expression of phage lysins. One example of this approach is the development of a tomato plant resistant to *Clavibacter michiganensis* infection, a common disease-causing Gram-positive bacterium, by the introduction of a phage lysin gene [8]. Here, the transgenic lysin-producing plant showed no disease symptoms after artificial infection, and tomatoes of normal quality and yield were obtained. While GMO regulations make their integration challenging, such applications using phage lysins to specifically target the pathogenic species may be beneficial over all-killing pesticides [119]. Cows have also been genetically engineered to produce lysostaphin in the mammary glands, successfully protecting against *S. aureus* infections [120]. In a final example, and currently the only attempted phage lysin production in a mammalian host, delivery of synthetic mRNA encoding endolysin Cpl-1 to human cells resulted in its endogenous expression and intracellular accumulation, as well as secretion [46]. The lysates and supernatant of the transfected cell lines were proven active against *S. pneumoniae* and the effect of N-linked glycosylation was investigated. Although translation to *in vivo* and further safety studies are needed, this mRNA-based lysin therapy allows for targeted delivery to the infected tissues and could avoid the need for repeated lysin dosing [121]. Finally, as the expressed mRNA accumulates intracellularly, pathogens residing here could also be targeted [46].

## Impact of the expression host on phage lysin yield and activity

It is conceivable that the lysin expression host has an impact on not only the expression yield, but also the activity of the lysin product. For example, host-associated modifications such as glycosylation described above. While these are arguably two major factors to take into account when attempting any lysin-based application, large-scale investigations of such host choice-dependent effects on lysin performance have not been conducted. Here, we examine the limited number of comparative studies.

### Impact on lysin yield

Table 1 presents examples of reported lysin production yields across different expression systems. With the exception of expression in *E. coli*, the listed examples are exhaustive, reflecting, to the best of our knowledge, all yields reported for lysin expression in their respective hosts. Generally, *E. coli* and yeast are known to deliver high protein yields, while expression in plants and mammalian cells typically result in lower productivities [19]. The lysin yields reported in Table 1 are consistent with these trends. For *E. coli*, a wide range of yields varying between the mg/L and the g/L scale, has been documented, largely due to its extensive use in research. A current maximum yield of 3.3 g/L was achieved by optimisation of the production process in a 20 L bioreactor [31]. In contrast, for other bacterial expression hosts such as lactobacilli, a yield of around 10 µg/L has been reported [37]. Yeast, particularly *H. polymorpha*, can also achieve substantial lysin yields, with the highest reported example being 43 mg/L [41]. Lysin expression in plants tends to be lower and is often measured per plant weight (wet or dry), making direct comparisons with other expression systems challenging. Important to note is that, while the aforementioned examples show some general trends, interpretation might be confounded by different factors. Several reported studies do not use purified expressed proteins, but rather use crude cell extracts or whole host cells in applications such as food fermentations. Additionally, the techniques for measuring protein concentration can vary. Finally, yields may be protein-dependent and are influenced by the specific expression machinery (e.g., promoter) used, which varies across the reported studies.

### Impact on lysin activity

Several papers compare the activity of specific lysins produced in yeast cells to their counterparts produced in common *E. coli* expression strains. In one study, the specific activities of *S. cerevisiae* BY4727-produced endolysins LysA and LysA2 were both lower (with a difference of up to 40% of specific activity) against three ethanol bacterial fermentation contaminants compared with their *E. coli* BL21(DE3)-produced counterparts, as measured in a TRA [118]. The authors speculate that this observation was due to differences in purity between expressions. However, further investigation using concentration- and purity-corrected proteins is needed to rule out any host-dependent effects. Another study expressed LysA2 in *P. pastoris* GS115 and the difference with *E. coli* BL21(DE3)-produced protein was again assessed by TRA [38]. While the authors stated that the activity was comparable, differences in the turbidity reduction profile were visible, suggesting a host influence. On the other hand, though no direct comparison in the same assay was performed, PVP-SE1gp146 lysin has shown similar activity when expressed in *H. polymorpha* NCYC495 (14.200.000 units/mM) and *E. coli* BL21(DE3)pLysS (13.613.960 units/mM) [41,122]. Variations in activity between *E. coli*- and plant-produced lysins have also been assessed. PlyCP41, an endolysin targeting *C. perfringens*, was expressed in both *E. coli* BL21(DE3) and *Nicotiana benthamiana* and its activity was evaluated in a plate lysis assay. Here, spotting of crude plant sap extract equivalent to 200 ng of lysin showed a similar clearing zone as 100 ng of the *E. coli*-produced variant [45]. Finally, expression and purification of a chimeric phage lysin in *N. benthamiana* showed 14% growth inhibition of *S. aureus* 305, while this was 68% using its *E. coli* BL21(DE3)-produced counterpart at the same concentration [93].

These studies suggest a possible host influence. More specifically, it seems that lysin expression in non-bacterial systems may be associated with a lower yield and activity. However, several confounding factors still prevent us from drawing definitive conclusions. These include differences in codon optimization, purification method and final purity, and concentration of the investigated proteins. Additionally, the specific activity assays used for comparison might influence the results. Assays based on plate lysis are less ideal in this regard, as the size of the observed halo zones is also dependent on factors beside activity such as protein diffusivity or substrate binding affinity [94]. Hence, future research comparing lysin yields and activity should be performed using purity- and concentration-corrected proteins in meaningful assays such as a growth inhibition assay or a TRA.

## Conclusion and outlook

In summary, we have explored the various factors associated with lysin production across different expression hosts and their subsequent effects on lysin yields and activity. For initial experiments, we would recommend *E. coli* hosts due to their widely available protocols and equipment. Beyond this, selecting an appropriate host is case-specific and should be guided by the intended application, considering the distinct features of the available expression systems. However, research into non-*E. coli* hosts for lysin expression remains sparse, emphasizing the need for further research to better understand how host selection influences lysin production and performance.

## Summary

- Phage lysins offer attractive properties for a wide variety of applications while the art of their recombinant production has not yet received much attention.
- We provide an overview of the available and used recombinant expression systems for the production of phage lysins with the aim of guiding both new and experienced researchers in their host choice.
- This choice is guided by several considerations such as endotoxin contamination, PTMs, protein solubility, and toxicity, but mainly the envisioned application.
- Our survey shows that there is room for further research exploring the suitability of expression hosts besides the common *E. coli* for phage lysin production.
- Future research comparing purity- and concentration-corrected proteins from different hosts or strains should provide insight into the impact of the host choice on the lysin activity.

## Abbreviations

CHO: Chinese hamster ovary
GIT: gastrointestinal tract
GRAS: Generally Regarded As Safe
IVTT: *in vitro* transcription-translation
LAB: lactic acid bacteria
LPS: lipopolysaccharide
MIC: minimum inhibitory concentration
PG: peptidoglycan
PTM: post-translational modification
TRA: turbidity reduction assay
